# Considerate motion imagination classification method using deep learning

**DOI:** 10.1371/journal.pone.0276526

**Published:** 2022-10-20

**Authors:** Zhaokun Yan, Xiangquan Yang, Yu Jin

**Affiliations:** 1 School of Martial Arts and Ethnic Traditional Sports, Tianjin Institute of Physical Education, Tianjin, China; 2 Tianjin Nankai District Experimental Kindergarten, Tianjin, China; University of Alicante: Universitat d’Alacant, SPAIN

## Abstract

In order to improve the classification accuracy of motion imagination, a considerate motion imagination classification method using deep learning is proposed. Specifically, based on a graph structure suitable for electroencephalography as input, the proposed model can accurately represent the distribution of electroencephalography electrodes in non-Euclidean space and fully consider the spatial correlation between electrodes. In addition, the spatial-spectral-temporal multi-dimensional feature information was extracted from the spatial-temporal graph representation and spatial-spectral graph representation transformed from the original electroencephalography signal using the dual branch architecture. Finally, the attention mechanism and global feature aggregation module were designed and combined with graph convolution to adaptively capture the dynamic correlation intensity and effective feature of electroencephalography signals in various dimensions. A series of contrast experiments and ablation experiments on several different public brain-computer interface datasets demonstrated that the excellence of proposed method. It is worth mentioning that, the proposed model is a general framework for the classification of electroencephalography signals, which is suitable for emotion recognition, sleep staging and other fields based on electroencephalography research. Moreover, the model has the potential to be applied in the medical field of motion imagination rehabilitation in real life.

## Introduction

Brain-computer interface is a widely studied human-computer interaction technology that creates a direct connection between the human brain and external devices, allowing people to communicate with the real world or manipulate external devices solely through neural activity in the brain [1]. Currently, there are many studies on brain-computer interfaces, such as motion imagination [2], emotion recognition [3] and sleep staging [4], among which motion imagination has attracted great attention in recent years. Motion imagination is the reproduction of specific actions related to human movement in the brain, but not accompanied by actual body movements. The correct recognition of neuronal activity in different motion imagination can lead to brain instructions that can help patients with severe motion neuron disease to control external equipment such as wheelchairs. Also, motion imagination classification is also an important support for rehabilitation training [5].

The brain-computer interface system includes both invasive and non-invasive methods to measure the neuronal activity of the brain. As one of the non-invasive methods, electroencephalography (EEG) is widely used because of its safety, reliability, comfort and convenience. The core problem in the study of motion imagination classification based on EEG signals is how to decode the EEG signals collected based on multiple electrodes into valid features and improve the accuracy of classification.

Many efforts have been made by scholars for the feature extraction of EEG signals. Early EEG classification methods extracted temporal features directly from waveforms, which could only be used for signals with significant temporal variation. Later, the recognition methods of motion imagination EEG signals based on artificial feature extraction can be roughly divided into two categories, namely spatial filtering method and EEG classification method based on the conversion of time domain to frequency domain. Representative methods of the former, such as common spatial pattern (CSP) [6], which extracted components of spatial distribution of each category from multi-channel EEG data and classify them. Ang et al. proposed filter bank common spatial pattern (FBCSP) [7], which added a feature selection algorithm on the basis of CSP to select distinguishable frequency band pairs and corresponding CSP features. Meanwhile, the representative methods of the latter include wavelet transform [8] and short-time Fourier transform [9]. However, these traditional methods only consider the spectral-temporal or spatial-temporal features, without taking into account the multidimensional or comprehensive (i.e., spatial dimension, temporal dimension and spectral dimension) features of EEG signals, and at the same time, the classification results are heavily dependent on expert experience.

Recently, deep learning technology has achieved great success in the fields of image processing and natural language processing by virtue of the advantages of automatic feature extraction. In order to solve the limitation of artificial feature extraction, many scholars have used deep learning technology to decode EEG signals, such as using two-dimensional or three-dimensional convolutional neural networks (CNNs) to automatically extract EEG signals for motion imagination classification. Schirrmeister et al. proposed a shallow convolutional network to automatically extract features directly from original EEG signals [10]. Zhao et al. proposed a multi-branch three-dimensional convolution model with three different convolution kernel sizes to extract features from the three-dimensional representation of EEG signals [11]. Wu et al. proposed a convolution neural network based on parallel multi-scale filter banks to extract EEG features [12]. However, most methods only involve the temporal and spatial characteristics of EEG signals, which, as mentioned above, are not considerate. Moreover, the distribution of EEG electrodes is not a natural Euclidean space or standard grid structure, and ordinary convolution cannot fully capture the spatial correlation between electrodes.

Since the electrodes of EEG are distributed in non-Euclidean space, graph convolution neural networks (GCNNs) are gradually used to classify motion imagination. Li et al. proposed an end-to-end spatial-temporal GCNN, which simultaneously captured the spatial-temporal features of EEG signals to identify different motion imagination [13]. Lun et al. [14] proposed a deep learning framework based on GCNN by combining the functional topological relationship of electrodes, so as to improve the decoding performance of motion imagination EEG signals. Sun et al. proposed an adaptive spatial-temporal GCNN, which can make full use of the characteristics of EEG signal in time domain and channel correlation in space domain [15]. In general, there are not many researches on the classification of motion imagination by graph convolution, and although these existing models have achieved improvement in classification performance, they do not take into account the association intensity of various dimensions that varies with different experiments due to the characteristics of EEG. It is still a challenge to represent, model and capture the dynamic correlation intensity of EEG signals in multiple dimensions of time, frequency and space.

To address the above challenges, this paper proposes a considerate attention-based multi-dimensional feature graph convolutional neural network (C-GCNN) to perform motion imagination classification. The main contributions of this paper are summarized as follows.

1. A graph structure is proposed for EEG signals that can accurately represent the non-Euclidean space of EEG electrode distribution and take into account the spatial correlation between electrodes.
2. A spatial-temporal and spatial-spectral dual branching architecture is proposed to simultaneously extract the feature information of EEG signals in three dimensions: temporal domain, spectral domain and spatial domain.
3. The C-GCNN model is designed to capture the dynamic correlation intensity of EEG signals in each dimension adaptively and extract EEG features effectively by combining attention mechanism and graph convolution for the first time.
4. Experiments are conducted on four publicly available brain-computer interface datasets to demonstrate that the proposed model outperforms other existing motion imagination classification methods.

## Materials and methods

### GCNN

A graph is made up of nodes and edges connecting two nodes. It is usually used to describe a particular relationship between things. Considering that the neighbor nodes in the graph structure are not fixed, traditional fixed-size and learnable convolution kernels cannot be used to extract graph node features. Therefore, scholars put forward the concept of graph convolution, which can be performed on a graph. There are two most commonly used construction methods: the spatial domain-based and the spectral domain-based. To construct graph convolution in the spatial domain is to apply the convolution kernel directly to the nodes and their neighborhoods on the graph [16]. However, because the neighborhood of each vertex is different, it needs to be processed for each vertex, so the calculation cost is high and the complexity is great. In the spectral domain, the convolution operation on the graph structure data can be realized by transforming the graph Laplian matrix to the spectral domain and solving the K-order truncation approximation of Chebyshev polynomials [17], so the computation cost is correspondingly low. based on this, spectral graph convolution is used in this paper to extract graph node features.

### C-GCNN

**Fig 1:**
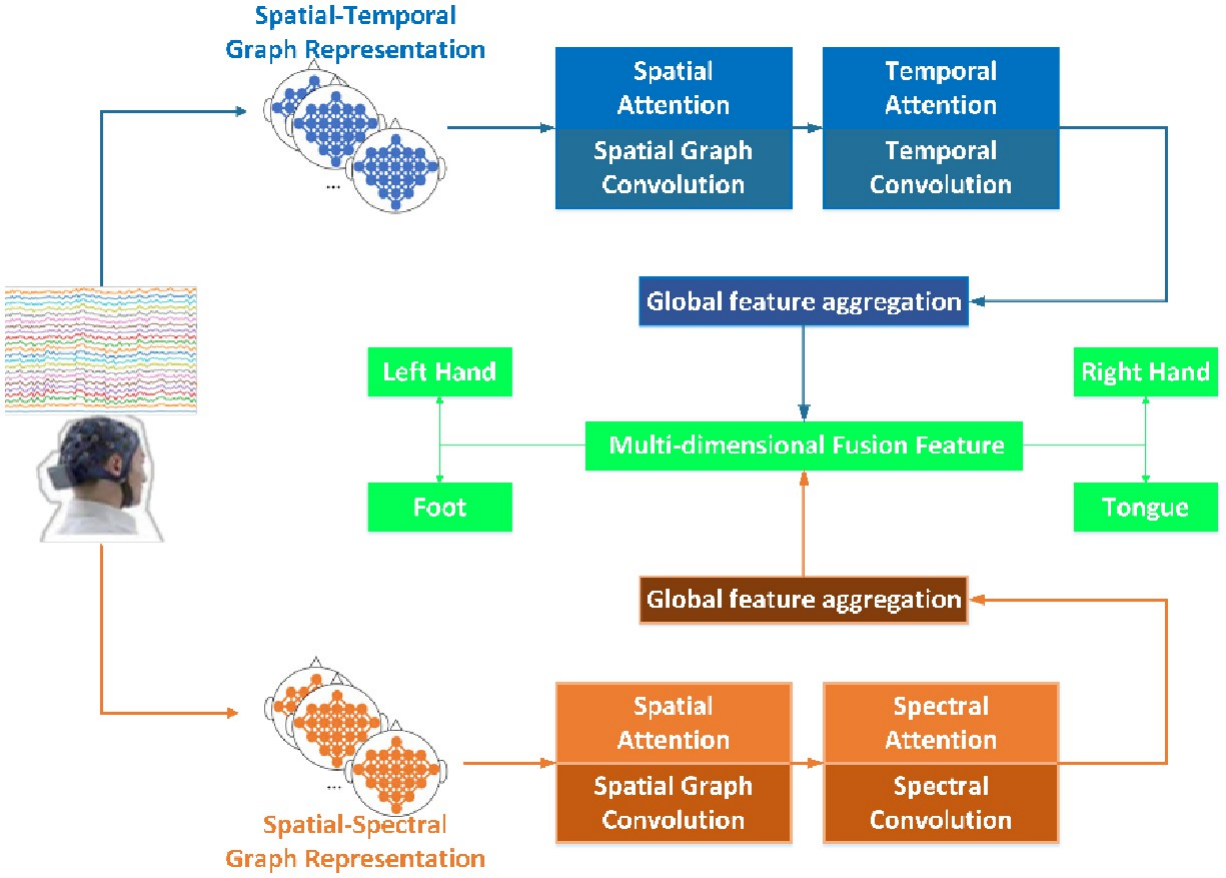
The flowchart.

In this paper, a novel C-GCNN model is proposed to decode and recognize the EEG signals generated by motion imagination. The overall framework of C-GCNN is shown in Fig 1.

As shown in the Fig 1, the raw EEG signals are converted into spatial-temporal graph representation and spatial-spectral graph representation based on the graph structure, and then fed into the network consisting of attention mechanism, graph convolution, temporal convolution, global feature aggregation and shortcut connection, respectively, and the outputs of the two branches are classified after feature fusion. The model as a whole consists of five parts, namely, the data transformation and its graph representation, an attention mechanism-based spatial graph convolution module, an attention mechanism-based temporal/spectral convolution module, global feature aggregation modules, and multidimensional feature fusion modules, which are described in detail in the following sections.

#### Data transformation and its graph representation

**Fig 2:**
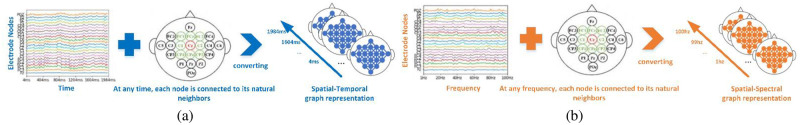
The conversion process of EEG signals. (a): The conversion process of Spatial-Temporal graph representation; (b): The conversion process of Spatial-Spectral graph representation.

Since the electrode node locations of EEG signals are not in standard Euclidean space, in order to accurately represent this property, a graph is constructed based on the natural spatial distribution of electrodes. The temporal and frequency domain information of the EEG signal is then mapped into the graph, and the specific conversion process is shown in Fig 2.

Spatial-temporal graph representation: The motion imagination raw EEG signal collected through multiple electrodes is a multiconductor signal defined as $X=\left[X_{1},X_{2},\cdots ,X_{N}\right]\in {\mathbb{R}}^{N\times T}$, where *N* is the number of electrode nodes of EEG and T is the duration of time. Each one-conductor EEG signal $X_{n\in \left[1,N\right]}=\left[S_{n}^{1},S_{n}^{1},\cdots ,S_{n}^{1}\right]\in {\mathbb{R}}^{T}$ is the one-dimensional temporal data.

**Fig 3:**
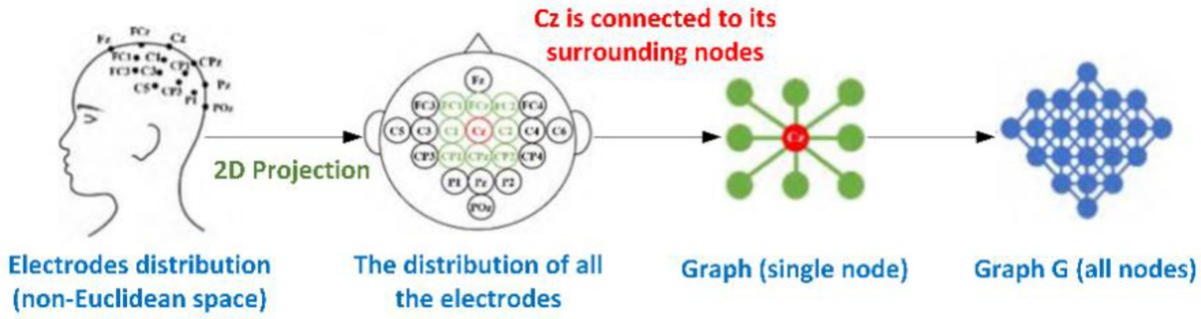
The construction process of graph.

In this paper, we construct a graph *G* applicable to EEG signals based on the natural spatial distribution of electrode nodes on the brain, and the construction process is shown in Fig 3. The graph is composed of nodes and edges, denoted as *G = (N*, *E)*, where *N* is the set of nodes of EEG electrodes and *E* is the set of edges. Considering that the voltage value of each electrode node is influenced by its surrounding voltage value, it is assumed in this paper that each node has 8 naturally adjacent nodes: upper, lower, left, right, top-left, top-right, bottom-left, bottom-right, and each node is assumed to be connected to itself. The set of edges is defined as *E = {N*_*i*_ *N*_*j*_ *| (i*, *j) ϵ H}*, where *H* is the set of naturally adjacent nodes. For the multiconductor EEG signal in the temporal domain, each time slice can form an undirected graph, and the entire time forms a spatial-temporal graph representation *x*^*st*^, which is used to describe the information of time in space.

Spatial-spectral graph representation: The time-frequency domain conversion is adopted for the original EEG signal to obtain frequency domain information. In this paper, the power spectral density (PSD) of different frequencies on each conductor is obtained using the Welch method to calculate each conductor *X*_*n*∈[1, *N*]_ [18], and it is denoted as $\left[f_{n}^{1},f_{n}^{1},\cdots ,f_{n}^{1}\right]\in {\mathbb{R}}^{N\times F}$, where *F* is the PSD feature length of each electrode. Again, based on the constructed graph structure, convert all PSD on each frequency into an undirected graph, and the graphs composed of all frequencies are combined to form a spatial-spectral graph representation, denoted as *x*^*ss*^, to describe the information of the spectrum in space.

#### Attention mechanism-based spatial graph convolution

In order to capture the dynamic association intensity between EEG nodes in the spatial domain adaptively, this paper designs an attention mechanism-based spatial graph convolution module, which consists of two parts: spatial attention mechanism and spatial graph convolution.

*Spatial attention mechanism*. In general, different motion imagination tasks trigger neuronal activity in different areas of the brain. Even when the same task is performed, the degree of activation in different regions varies from person to person. Therefore, the intensity of the association between brain nodes is dynamic. Inspired by the self-attentive mechanism [19], this paper designs a spatial attention mechanism to capture this dynamic association intensity adaptively, which is computed as follows:

Since the structure of the spatial-temporal branch and the spatial-spectral branch are identical, the time-space branch is described here as an example. The input of the module is $x^{st}\in {\mathbb{R}}^{N\times T\times C}$, where *C* is the number of channels, and the module adaptively calculates $a_{s}\in {\mathbb{R}}^{N\times N}$ according to *x*^*st*^:

$$a_{s}=\sigma (x^{st}W_{s_{1}}+b_{s_{1}})\sigma (W_{s_{2}}x^{st}+b_{s_{2}})$$ (1)

where *σ* is the activation function Tanh, $W_{s_{1}},W_{s_{2}}\in {\mathbb{R}}^{C}$ is the weight matrix, $b_{s_{1}}\in {\mathbb{R}}^{N\times T}$ and $b_{s_{2}}\in {\mathbb{R}}^{T\times N}$ are the deviations.

Typically, the *a*_*s*_ is normalized using the Softmax normalization function. However, although Softmax can guarantee that different electrodes are separable from each other, it cannot achieve the effect of intra-region compactness and inter-region separation. Therefore, in this paper, we propose to compute the spatial attention matrix by L2 normalization of *a*_*s*_. L2 normalization can make the feature vectors as compact as possible within regions and as separated as possible between regions, which can better improve the model performance. The spatial attention matrix $a_{s}^{\prime }\in {\mathbb{R}}^{N\times N}$ is defined as

$$a_{s}^{\prime }=\frac{a_{s}^{i,j}}{{\left\|a_{s}^{i,j}\right\|}_{2}}$$ (2)

where the element $a_{s}^{i,j}$ denotes the magnitude of the association intensity of node *i* and node *j*.

*Spatial graph convolution*. In order to reduce the computational cost, this paper uses spectral graph convolution to extract the spatial features of EEG signals by performing a convolution operation on the graph structure data after being adjusted by the spatial attention mechanism. The specific process is as follows.

Based on the constructed graph, calculate the adjacency matrix $A_{i,j}\in {\mathbb{R}}^{N\times N}$:

$$A_{i,j}=\left\{\begin{array}{ll} 1 & N_{i}N_{j}\in E\\ 0 & N_{i}N_{j}\notin E \end{array}\right.$$ (3)

The corresponding Laplacian matrix is denoted as $L=D-A\in {\mathbb{R}}^{N\times N}$, where the degree matrix $D\in {\mathbb{R}}^{N\times N}$ is the diagonal matrix *D*_*ii*_ = ∑_*j*_ *A*_*ij*_, consisting of the degrees of the graph nodes. The eigen decomposition after regularization of the Laplace matrix yields $L=I_{E}-D^{-\frac{1}{2}}AD^{-\frac{1}{2}}=U\Lambda U^{T}$, where *I*_*E*_ is the unit matrix, U is the eigenvector matrix, and Λ is the diagonal matrix of eigenvalues.

Taking the EEG nodal graph $x=x_{t}^{st}$ at moment *t* as an example, the spectral graph convolution on the graph can be defined as the product with the Fourier domain filter *g*_*θ*_ = *diag*(*θ*), and *g*_*θ*_ can be interpreted as a function about the eigenvalue of *L*, i.e., *g*_*θ*_ (Λ), and here the truncated expansion of the K-order Chebyshev polynomial *T*_*k*_(*x*) is used to approximate *g*_*θ*_ (Λ). Thus, the spectral graph convolution is computed as

$$g_{\theta }\times x\approx \sum_{k=0}^{K}\theta_{k}^{\prime }T_{k}(\tilde{L})x$$ (4)

where $\theta^{\prime }\in {\mathbb{R}}^{K}$ is a vector of Chebyshev coefficients, and the recursive definition of the Chebyshev polynomial is: *T*_*k*_(*x*) = 2*xT*_*k*−1_(*x*) − *T*_*k*−2_(*x*), *T*_0_(*x*) = 1, *T*_1_(*x*) = *x*.

In this paper, each order of Chebyshev polynomial $T_{k}(\tilde{L})\in {\mathbb{R}}^{N\times N}$ is multiplied with the computed spatial attention matrix $a_{s}^{\prime }$ and the ReLU function is used as the activation function. Thus, the output $x_{1}^{st}\in {\mathbb{R}}^{N\times T\times C_{1}}$ of the spatial graph convolution based on the spatial attention mechanism is defined as:

$$x_{1}^{st}=\sigma (g_{\theta }\times x)\approx \sigma \left(\sum_{k=0}^{K}\theta_{k}^{\prime }(T_{k}(\tilde{L})⊗a_{s}^{\prime })x\right)$$ (5)

where *σ* represents the activation function and ⊗ represents the multiplication of corresponding elements.

#### Attention mechanism-based temporal / spectral convolution

In order to extract the features of spectral-temporal domains in EEG and capture the dynamic correlation intensity between time and time and between spectrum and spectrum in EEG adaptively, this paper designs an attention mechanism-based temporal / spectral convolution module, including temporal / spectral attention mechanism and temporal / spectral convolution.

*Temporal / spectral attention mechanism*. The EEG signal is the multiple time series that varies with time, and there is a certain interplay and dependence of its voltage values at different moments. Likewise, the frequency spectral density between adjacent frequencies also affects and depends on each other. Therefore, this paper designs a temporal / spectral attention mechanism to capture this dynamically changing correlation adaptively. In particular, proposed temporal attention and spectral attention act on two branches separately, but with the same structure, and the spatial-temporal branch is still described as an example. The relevant specific computational procedure is as follows:

The input of this module is the output of the previous module $x_{1}^{st}\in {\mathbb{R}}^{N\times T\times C_{1}}$, and based on $x_{1}^{st}$, the attention mechanism first adaptively computes $a_{t}\in {\mathbb{R}}^{T\times T}$:

$$a_{t}=\sigma (x_{1}^{st}W_{t_{1}}+b_{t_{1}})\sigma (W_{t_{2}}x_{1}^{st}+b_{t_{2}})$$ (6)

where *σ* is the activation function Tanh, $W_{s_{1}},W_{s_{2}}\in {\mathbb{R}}^{C}$ is the weight matrix, $b_{s_{1}}\in {\mathbb{R}}^{N\times T}$ and $b_{s_{2}}\in {\mathbb{R}}^{T\times N}$ are the deviations.

Secondly, L2 normalization of *a*_*t*_ yields the temporal attention matrix $a_{t}^{\prime }\in {\mathbb{R}}^{T\times T}$:

$$a_{t}^{\prime }=\frac{a_{t}^{i,j}}{{\left\|a_{t}^{i,j}\right\|}_{2}}$$ (7)

where $a_{t}^{i,j}$ denotes the intensity of the association between timing *i* and timing *j*.

*Temporal / spectral convolution*. After the adjustment of the temporal / spectral attention mechanism, this paper chooses to use the standard convolution in two dimensions to learn the temporal dependence as well as the spectral dependence, respectively. Although deep neural networks have good learning representation capability, for EEG analysis, it is not the deeper the network, the better the results. Therefore, one convolution layer has been able to capture the temporal and spectral features on each node very well. In this paper, the specific structure of the temporal / spectral convolution is shown in Table 1.

**Table 1:** The structure of temporal / spectral convolution.

Layer	KernelSize/Stride	Kernel	Activation
temporal / spectral convolution	(1,5)/(1,1)	64	ReLU

After the computation of the temporal convolution module based on the attention mechanism, we can obtain $x_{2}^{st}\in {\mathbb{R}}^{N_{2}\times T_{2}\times C_{2}}$, i.e.,

$$x_{2}^{st}=\sigma (W_{2}(x_{2}^{st}a_{t}^{\prime })+b_{2})$$ (8)

where W_2_ and *b*_*2*_ are the weights and biases learned by temporal convolution, respectively.

#### Global feature aggregation

In order to globally consider the feature information between all nodes and the feature information between all time/spectrum, a global feature aggregation module is designed to aggregate spatial global features and temporal / spectral global features through two convolutional layers, respectively. Moreover, the nonlinear function ReLU between the convolutional layers can also make the model learn more complex functions, and thus increase the model complexity.

Again, using the spatial-temporal branch as an example, first the features between all nodes are aggregated to obtain the global spatial features $x_{3}^{st}\in {\mathbb{R}}^{1\times T_{2}\times C_{2}}$:

$$x_{3}^{st}=\sigma (W_{3}x_{2}^{st}+b_{3})$$ (9)

where W_3_ and *b*_*3*_ are the weights and deviations of the global spatial aggregation, respectively.

Then aggregating all the features in temporal domain to get the global temporal feature $x_{4}^{st}\in {\mathbb{R}}^{1\times 1\times C_{2}}$:

$$x_{4}^{st}=\sigma (W_{4}x_{3}^{st}+b_{4})$$ (10)

where W_4_ and *b*_*4*_ are the weights and deviations of the global temporal aggregation, respectively.

The structure of the global feature aggregation module is set as shown in Table 2.

**Table 2:** The structure of global feature aggregation.

Layer	KernelSize/Stride	Kernel	Activation
Global spatial aggregation	(N2, 1)/1	64	ReLU
Global spectral-temporal aggregation	(1, T2)/1	64	ReLU

#### Multidimensional feature fusion

The spatial-temporal branch and the spatial-spectral branch added with the input through a series of feature extraction and aggregation operations by shortcut connections to form the spatial-temporal feature $\tilde{Y_{st}}\in {\mathbb{R}}^{C_{2}}$ and the spatial-spectral feature $\tilde{Y_{ss}}\in {\mathbb{R}}^{C_{2}}$, respectively. They are concatenated and fed into the fully connected layer to form a fused feature vector $\tilde{Y}$. This feature vector fuses all the features contained in the temporal, spectral and spatial dimensions of the EEG signal and can provide comprehensive, considerate and valuable feature information for classification. Finally, $\tilde{Y}$ is normalized by Softmax to perform the final classification.

$$\tilde{Y}=W_{st}{\tilde{Y}}_{st}+W_{ss}{\tilde{Y}}_{ss}$$ (11)

where W_st_ and W_ss_ are learning parameters reflecting the different degrees of influence of the two branches on the motion imagination classification.

## Results

### The dataset

In order to demonstrate the effectiveness of proposed method, four publicly available brain-computer interface datasets used in this paper, i.e., the BCI Competition IV dataset 2a (BCICIV-2a), the BCI Competition III dataset 3a (BCIC III -3a), the large EEG dataset HaLT (HaLT) and the AHU-MIEEG dataset (AHU-MIEEG).

BCICIV-2a [20]: The dataset contains EEG signals from 9 subjects doing different motion imagination tasks, namely imagining 4 types of motion imagination tasks: left hand, right hand, foot and tongue movements. The EEG signals are recorded using 22 electrodes and the sampling frequency of 250 Hz. A total of two sets of experiments are performed for each individual on different days. Each set of experiments consists of 288 motion imagination sessions, with an average of 72 sessions for each type of motion imagination.

BCIC III -3a [21]: The dataset consists of 3 subjects, the first of whom performs 360 motion imagination sessions and the others 240 sessions. There are 4 types of motion imagination tasks: left hand, right hand, foot and tongue. The EEG signals are collected using 60 EEG electrodes and recorded at the sampling frequency of 250 Hz.

HaLT [22]: Given the relatively early date of all BCI competitions, a large public EEG signal dataset released in recent years is also selected for this paper. The HaLT dataset is a subset of the "Large EEG Motion Imagination Dataset for Brain-Computer Interface EEG". It contains 12 subjects with 6 types of motion imagination tasks: left hand, right hand, left leg, right leg, tongue, and stillness. EEG signals are recorded at the sampling frequency of 200 Hz and 19 EEG electrodes. A total of 29 experiments are included in the dataset, with approximately 900 motion imagination sessions in each experiment, including different imagination tasks.

AHU-MIEEG [23]: The dataset is a publicly available motion imagination EEG data set from Anhui University, of which 10 subjects are selected for this experiment. The data are collected by Neuroscan amplifier with 26 electrodes and 250Hz sampling frequency, and there are3 types of motion imagination tasks: left hand, right hand and foot. Each subject performs the experiment on a different day, and each experiment consists of approximately 75 motion imagination sessions, with an average of 25 sessions of each type of motion imagination.

### Evaluation index

In this paper, accuracy and Kappa coefficient, which commonly used in motion imagination classification, are used as the evaluation index of the proposed model. Among them, accuracy is the proportion of motion imagination being correctly classified, i.e., the ratio of the number of correctly classified samples to the total number of samples, and the Kappa coefficient is calculated by the following formula,

$$\kappa =\frac{p_{o}-p_{e}}{1-p_{e}}$$ (12)

in which $p_{e}=\frac{a_{1}\times b_{1}+a_{2}\times b_{2}+\cdots a_{m}\times b_{m}}{n\times n}$, where *a*_*m*_ represents the number of true samples of the m-th class, *b*_*m*_ represents the number of predicted number of samples, n represents the total number of samples and *p*_*o*_ is the overall classification accuracy.

### Experiment settings

In this paper, all sets of experimental data for each subject are combined and the proposed model is validated using a 5-fold cross-validation method, finally the obtained results are averaged. The model is optimized using the Adam optimizer algorithm to minimize the cross-entropy loss function during the training process, and the learning rate is set to 0.001. The batch size is set to 64, i.e., 64 samples are selected for model optimization each time. In the graph representation, the time length T and frequency length F are both set to 100, and the K in the Chebyshev polynomial is set to 3.

All experiments of this paper are implemented in Python, where TensorFlow and Keras frameworks are used for the model part, and the models are trained and tested on a GPU server. Table 3 gives a detailed description of the hardware and software environments used in the experiments.

**Table 3:** Details of the experimental environment.

environments	versions
Operating System	Ubuntu 16.04.2 LTS
memory	128G
CPU	Intel(R)Xeon(R)CPU
	E5-2683 v3@2.00GHz
GPU	Tesla K80
CUDA	10.1
cuDNN	7.5.1
Python	3.6.8
TensorFlow	1.13.1
Keras	2.1.6

### Data augmentation

In the field of deep learning, the amount of training data is crucial for improving classification accuracy. Since motion imagination experiments are time-consuming and complex, it is not possible to obtain a large amount of EEG signals. Therefore, this paper uses data augmentation to generate more training data from the original EEG signals. In the BCICIV-2a and BCICIII-3a datasets, each motion imagination task contains 3s of EEG signal data. In this paper, we choose a common data enhancement method in EEG signal, i.e., sliding window, and set the window size to 2s and the sliding step to 0.32s, and enhance the EEG data to 4 times of the original. In the HaLT dataset, each motion imagination task contains only 1s of EEG signal data, and considering the short duration of the tasks, this paper adopts the method of adding white noise for its data enhancement.

### Benchmark methods

In order to verify the superiority of C-GCNN on motion imagination classification task, some excellent traditional and deep learning methods in motion imagination classification research are selected as benchmark methods to compare with C-GCNN in this paper, and the related benchmark methods are described as follows:

FBCSP [7]: a spatial filtering method that extracts the spatially distributed components of each type from a multichannel EEG signal and then classifies them using linear discriminant analysis.

Shallow-ConvNet [24]: a shallow convolutional network that uses two convolutional layers as temporal convolution and spatial filter, respectively, to extract the features of the original EEG signal.

EEGNet [25]: a compact CNN that uses depthwise separable convolutions to build EEG classification models.

Multi-branch-3D [26]: a multi-branch 3D convolutional model with three different convolutional kernel sizes to extract spatial-temporal features from the 3D representation of EEG signals.

MSFBCNN [27]: a parallel multiscale filter bank CNN to extract temporal and spatial features from EEG.

CNN-LSTM [28]: a one-versus-rest filter bank common spatial pattern (OVR-FBCSP), CNN and long short-term memory (LSTM) [29] -based hybrid deep neural network to decode the EEG signals of motion imagination.

### Contrast experiments

In order to verify the effectiveness of C-GCNN in the motion imagination classification task, it is compared with the most representative benchmark methods on four datasets. The same data preprocessing and 5-fold cross-validation are applied to all benchmark methods. Tables 4–7 show the classification accuracy and Kappa coefficients of the different methods in the BCICIV-2a, BCICIII-3a, HaLT, and AHU-MIEEG datasets, respectively. Since the proposed method is based on a subject-specific motion imagination classification study, the classification accuracy and Kappa coefficients are calculated for each individual and averaged across all individuals in each dataset.

**Table 4:** The contrast results of different methods in BCICIV-2a dataset.

Subject	Metric	FBCSP	Shallow CovNet	EEGNet	Multi-branch-3D	MSFB CNN	CNN-LSTM	C-GCNN
A01	Acc	0.7344	0.8333	0.8333	0.8122	0.8160	0.8741	**0.8898**
	Kappa	0.6457	0.7774	0.7773	0.7496	0.7541	0.8319	**0.8529**
A02	Acc	0.5608	0.6775	0.6380	0.6795	0.6415	0.7739	**0.8233**
	Kappa	0.4141	0.5692	0.5168	0.5727	0.5220	0.6977	**0.7644**
A03	Acc	0.8042	0.8741	0.8876	0.8409	0.8698	0.9073	**0.9093**
	Kappa	0.7388	0.8320	0.8498	0.7878	0.8260	0.8430	**0.8790**
A04	Acc	0.5768	0.7400	0.6241	0.6569	0.6814	0.8277	**0.8359**
	Kappa	0.4359	0.6533	0.4992	0.5425	0.5742	0.7700	**0.7810**
A05	Acc	0.5738	0.6341	0.5872	0.7058	0.7127	0.7289	**0.7335**
	Kappa	0.4313	0.5119	0.4497	0.6078	0.6158	0.6301	**0.6445**
A06	Acc	0.4948	0.7687	0.5851	0.6757	0.6337	0.8251	**0.8355**
	Kappa	0.3263	0.6917	0.4462	0.5676	0.5111	0.7665	**0.7805**
A07	Acc	0.8125	0.8646	0.8481	0.8587	0.9054	0.8958	**0.9171**
	Kappa	0.7497	0.8193	0.7972	0.8116	0.8736	0.7940	**0.8894**
A08	Acc	0.7352	0.8598	0.8212	0.8494	0.7787	0.8517	**0.8780**
	Kappa	0.6463	0.8128	0.7908	0.7991	0.7047	0.7684	**0.8373**
A09	Acc	0.6636	0.8238	0.7830	0.7810	0.7031	0.8891	**0.8945**
	Kappa	0.5514	0.7647	0.7106	0.7080	0.6035	0.8176	**0.8593**
Mean	Acc	0.6618	0.7862	0.7342	0.7622	0.7491	0.8415	**0.8574**
	Kappa	0.5488	0.7147	0.6453	0.6830	0.6650	0.7688	**0.8098**

**Table 5:** The contrast results of different methods in BCICIII-3a dataset.

Subject	Metric	FBCSP	Shallow CovNet	EEGNet	Multi-branch-3D	MSFB CNN	CNN-LSTM	C-GCNN
K3b	Acc	0.9139	0.9674	0.9625	0.9481	0.9636	0.9118	**0.9840**
	Kappa	0.8848	0.9564	0.9499	0.9309	0.9517	0.8821	**0.8797**
K6b	Acc	0.6792	0.7698	0.7052	0.7528	0.7854	0.8833	**0.9229**
	Kappa	0.5721	0.6911	0.6037	0.6704	0.7135	0.8406	**0.8967**
L1b	Acc	0.8031	0.8292	0.8021	0.8078	0.8229	0.9167	**0.9573**
	Kappa	0.7369	0.7710	0.7354	0.7437	0.7631	0.8868	**0.9430**
Mean	Acc	0.7987	0.8554	0.8233	0.8363	0.8574	0.9039	**0.9547**
	Kappa	0.7313	0.8062	0.7642	0.7817	0.8094	0.8699	**0.9394**

**Table 6:** The contrast results of different methods in HaLT dataset.

Subject	Metric	FBCSP	Shallow CovNet	EEGNet	Multi-branch-3D	MSFB CNN	CNN-LSTM	C-GCNN
1	Acc	0.4765	0.8476	0.8740	0.8672	0.8999	0.7073	**0.9114**
	Kappa	0.3720	0.8169	0.8486	0.8406	0.8798	0.6487	**0.8935**
2	Acc	0.2863	0.6752	0.6722	0.7337	0.7837	0.6970	**0.8313**
	Kappa	0.1438	0.6100	0.6065	0.6804	0.7363	0.5964	**0.7974**
3	Acc	0.3682	0.7933	0.8236	0.7988	0.8209	0.6898	**0.8353**
	Kappa	0.2417	0.7516	0.7881	0.7585	0.7807	0.6074	**0.8023**
4	Acc	0.3599	0.7483	0.7694	0.7699	0.8149	0.7716	**0.8271**
	Kappa	0.2315	0.6978	0.7231	0.7237	0.7777	0.7053	**0.7924**
5	Acc	0.2998	0.7037	0.7032	0.7412	0.7904	0.7586	**0.7961**
	Kappa	0.1590	0.6444	0.6437	0.6893	0.7482	0.7103	**0.7552**
6	Acc	0.3830	0.8458	**0.8933**	0.8549	0.8914	0.8787	0.8781
	Kappa	0.2595	0.8149	**0.8719**	0.8258	0.8695	0.8341	0.8536
7	Acc	0.2210	0.3844	0.4346	0.3999	0.4681	0.5080	**0.5159**
	Kappa	0.0646	0.2610	0.3213	0.2797	0.3614	0.4121	**0.4190**
8	Acc	0.2135	0.3916	0.4425	0.3886	0.4541	0.4918	**0.4937**
	Kappa	0.0569	0.2695	0.3315	0.2660	0.3409	0.3902	**0.3920**
9	Acc	0.7272	0.9894	0.9884	0.9884	0.9884	0.6920	**0.9900**
	Kappa	0.6717	0.9873	0.9860	0.9860	0.9860	0.5837	**0.9879**
10	Acc	0.4180	0.7968	0.8103	0.8174	0.8534	0.5862	**0.8537**
	Kappa	0.3012	0.7559	0.7720	0.7808	0.8240	0.4822	**0.8243**
11	Acc	0.5961	0.9559	0.9535	0.9283	0.9485	0.7294	**0.9561**
	Kappa	0.5150	0.9470	0.9441	0.9139	0.9382	0.6351	**0.9473**
12	Acc	0.4443	0.8296	0.8493	0.8590	0.8945	0.8521	**0.9048**
	Kappa	0.3329	0.7954	0.8190	0.8307	0.8733	0.7821	**0.8856**
Mean	Acc	0.3995	0.7468	0.7679	0.7623	0.8007	0.6969	**0.8161**
	Kappa	0.2792	0.6960	0.7213	0.7146	0.7597	0.6156	**0.7792**

**Table 7:** The contrast results of different methods in AHU-MIEEG dataset.

Subject	Metric	FBCSP	Shallow CovNet	EEGNet	Multi-branch-3D	MSFB CNN	CNN-LSTM	C-GCNN
S1	Acc	0.7953	0.8185	0.5396	0.5069	0.7904	0.8193	**0.8200**
	Kappa	0.6927	0.7277	0.2494	0.2007	0.6851	0.7295	**0.7297**
S2	Acc	0.4384	0.3930	0.5372	0.5204	0.4121	0.5807	**0.5932**
	Kappa	0.1580	0.0921	0.2472	0.2210	0.1187	0.3730	**0.3899**
S3	Acc	0.8617	0.8026	0.7610	0.7771	0.9099	0.8868	**0.9208**
	Kappa	0.7926	0.7042	0.5817	0.6059	0.8649	0.8298	**0.8809**
S4	Acc	0.6895	0.7232	0.5414	0.5375	0.5534	**0.8143**	0.8081
	Kappa	0.5340	0.5849	0.2527	0.2466	0.3361	**0.7212**	0.7119
S5	Acc	0.3602	0.5263	0.6562	0.6136	0.6872	0.7087	**0.7345**
	Kappa	0.0389	0.2889	0.4247	0.3604	0.5308	0.5655	**0.6020**
S6	Acc	0.5339	0.4696	0.5656	0.5890	0.7053	0.6801	**0.7083**
	Kappa	0.3009	0.2044	0.2905	0.3227	0.5576	0.5225	**0.5627**
S7	Acc	0.5359	0.4354	0.5098	0.5629	0.4483	0.6103	**0.6162**
	Kappa	0.3027	0.1561	0.2106	0.2838	0.1733	0.4296	**0.4296**
S8	Acc	0.4018	0.5150	0.5058	0.4997	0.5405	0.5469	**0.5645**
	Kappa	0.1014	0.2713	0.1995	0.1896	0.3106	0.3208	**0.3472**
S9	Acc	0.4662	0.6268	0.5169	0.5527	0.7602	0.7431	**0.7764**
	Kappa	0.1956	0.4385	0.2136	0.2696	0.6397	0.6176	**0.6646**
S10	Acc	0.3481	0.3264	0.4440	0.4495	0.4163	0.4380	**0.4519**
	Kappa	0.0251	0.0290	0.1711	0.1310	0.1269	0.1677	**0.1779**
Mean	Acc	0.5431	0.5637	0.5577	0.5609	0.6224	0.6828	**0.6994**
	Kappa	0.3142	0.3497	0.2861	0.2831	0.4344	0.5277	**0.5492**

From the tables, we can see that as a spatial filter-based traditional EEG classification method, FBCSP only considers spatial information and ignores the discriminative features about time and frequency information, so the classification results are poor. In contrast, methods such as Shallow-ConvNet, EEGNet and MSFBCNN extract temporal and spatial features from EEG by designing different types of 2D convolutions. Multi-branch-3D uses 3D convolution kernels of different sizes to extract spatial and temporal features simultaneously. CNN-LSTM combines the various traditional deep learning-based methods such as FBCSP, CNN and LSTM to extract spatial and temporal features. These methods take into account the features of both temporal and spatial dimensions of EEG signals, so the classification performance is better than that of FBCSP.

The C-GCNN proposed in this paper has the best performance in terms of average accuracy and average Kappa coefficient over the four datasets compared with all benchmark methods. This is because C-GCNN extracts spatial-spectral-temporal features simultaneously based on the graph representation suitable for EEG signals, and obtains more accurate and comprehensive/considerate feature information. Moreover, C-GCNN also utilizes an attention mechanism to adaptively capture the dynamic correlation intensity of EEG signals in different dimensions, which makes the model more robust. In the results of the single-subject experiments, EEGNet achieves the best classification results on the HaLT dataset on subject 6 and CNN-LSTM on subject 4 in the dataset AHU-MIEEG. This may be due to the individual differences in EEG signals generated by motion imagination and the depthwise separable convolution of EEGNet and the hybrid network of CNN-LSTM better capture the feature information of these two subjects’ feature information. In comparison, C-GCNN, although not capture the most suitable EEG features for these 2 subjects, but also achieves excellent classification results. In addition, C-GCNN obtains the best classification performance on all other subjects. In general, C-GCNN can improve the classification performance of motion imagination for most subjects and ensure that the average classification result in each dataset is optimal.

### Ablation experiments

In order to further investigate the roles of different modules in C-GCNN, five variants about C-GCNN are designed in this paper, and the differences between these variants are described as follows:

1. Spatial-temporal graph convolution: This variant has only the spatial-temporal branch of C-GCNN, which includes spatial graph convolution and temporal convolution.
2. Spatial-spectral graph convolution: This variant has only the spatial-spectral branch of C-GCNN, and only spatial graph convolution and spectral convolution are included in this branch.
3. Dual branch: This variant includes both the spatial-temporal branch, the spatial-spectral branch and the feature fusion of the last two branches of C-GCNN.
4. ADD Global feature aggregation: Based on variant 3(i.e., dual branch), the global feature aggregation modules are added.
5. ADD Attention mechanism: Based on variant 4, this variant adds attention mechanisms, namely spatial attention and temporal/spectral attention.

**Fig 4:**
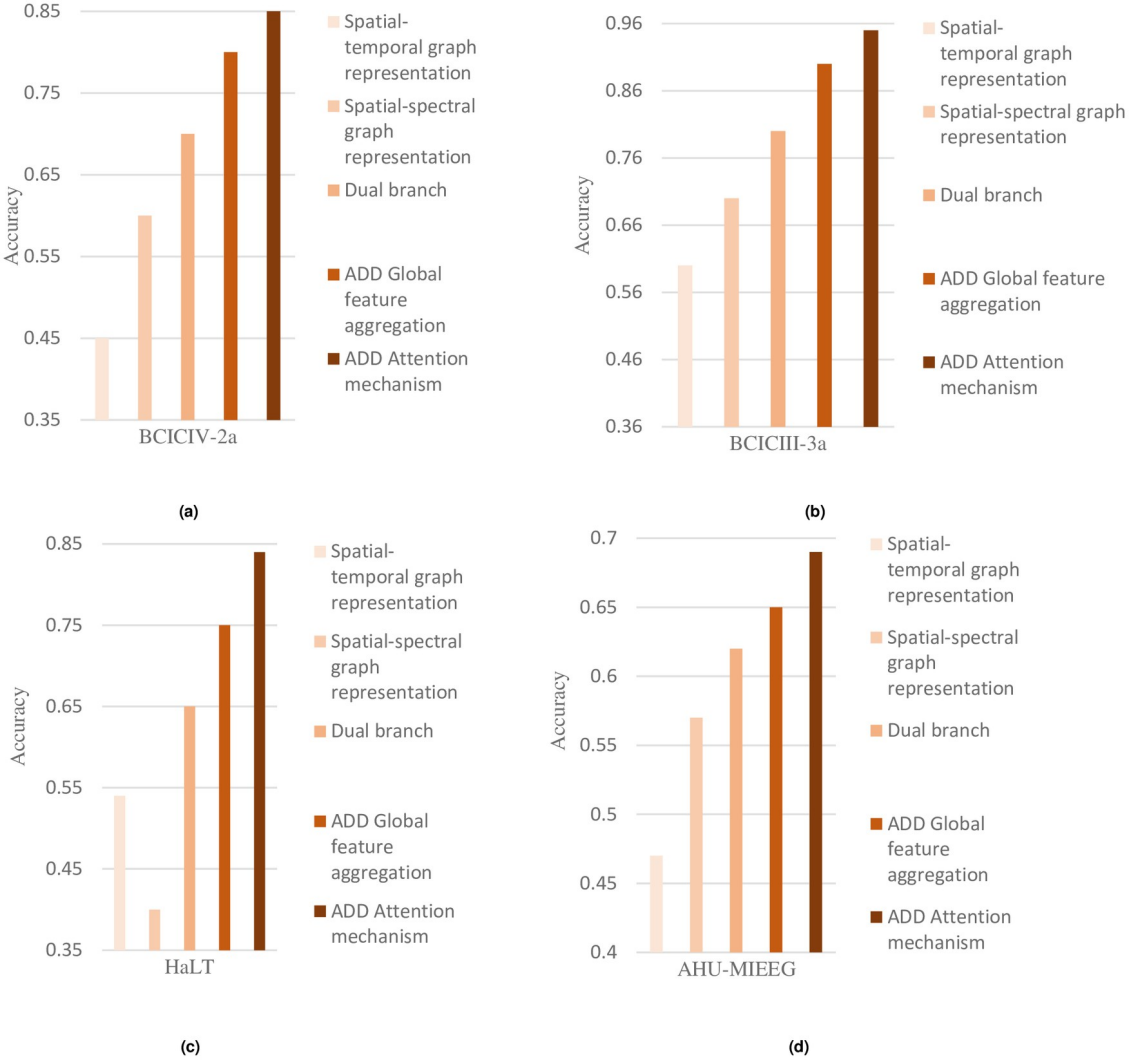
The ablation results of different variants. (a) BCICIV-2a dataset. (b) BCICIII-3a dataset. (c) HaLT dataset. (d) AHU-MIEEG dataset.

Fig 4 shows the comparison of the average classification accuracy of all 5 variants of the model in the datasets BCICIV-2a, BCICIII-3a, HaLT, and AHU-MIEEG. It can be seen that if extracting features in the spatial-spectral-temporal dimensions of EEG can provide more and richer discriminative features than extracting temporal features or spatial-spectral features alone, and thus obtain better classification performance. Moreover, the global feature aggregation module and attention mechanism designed in this paper can improve the classification accuracy of the model for different motion imagination varying degrees. In conclusion, it can be proved that each module of the proposed C-GCNN model is effective and can improve the performance of motion imagination classification.

## Conclusions

As an important application of brain-computer interface, motion imagination is an important support for sports rehabilitation training. Because the distribution of electroencephalography electrodes is not a natural Euclidean space, it is a great challenge to accurately classify motion imagination. In addition, the existing methods only consider the information of a certain dimension or two dimensions in electroencephalography signals, and cannot considerately capture the inherent characteristics of electroencephalography signals in spatial-spectral-temporal aspect. At the same time, the dynamic correlation intensity of each dimension of electroencephalography affected the robustness of classification. To solve the above problems, this paper proposes a considerate attention-based multi-dimensional feature graph convolutional neural network (C-GCNN) integrating the attention mechanism. Firstly, a graph structure is designed according to the non-Euclidean spatial characteristics of electrode node distribution to fully represent the spatial correlation between electrodes. Secondly, the spatial-temporal and the spatial-spectral architectures are proposed to represent the information of electroencephalography in spatial-spectral-temporal domain simultaneously. Finally, the spatial representation, temporal dependence and spectral dependence of electroencephalography signals are learned from graph representation by integrating attention mechanism, graph convolution and temporal / spectral convolution, and the dynamic correlation intensity of each dimension is captured adaptively. A series of contrast experiments and ablation experiments on several different public brain-computer interface datasets show that the proposed C-GCNN model achieved some improvement in motion imagination classification task compared with other benchmark methods. Although the proposed method has some unique advantages, there are still some problems that need to be studied at a turn. For example, the current research is aimed at each subject, so how to propose a more universal algorithm for cross-subject research needs further discussion and analysis.
